# Ossification of the Superior Lobe of the Right Lung

**Published:** 1849-09

**Authors:** 

**Affiliations:** De Pere, Wis.; De Pere, Wis.


### ARTICLE VII.

*Ossification of a part of the Superior Lobe of the Right Lung, with a Pathological Specimen.* From Drs. WILLIAMS & JOHNSON of De Pere, Wis.

MESSRS. EDITORS:—

By the bearer we send you a specimen of diseased lung, which you may call encysted tubercle, osseous composition, or any other name your sense of propriety may dictate, after examining it. Hunting for the history of a similar case, we find Laennec, in treating on encysted tubercles, says: "I have myself, never seen these cysts, whether primitive or secondary, become ossified; this morbid state must therefore be rare; but I have in my possession a cyst of the size of a hen's egg, converted into a bony substance, which was found in the lungs of a subject, who seemed to have died, as far as I could learn, of phthisis." This isolated case seems to comprise the only knowledge of that celebrated writer on diseases of the kind here presented. Mackintosh speaks of it as an osseous composition of rare occurrence, while a large majority of writers and lecturers do not mention it.

About the middle of Jan., 1849, Dr. Williams was called to see Mr. A., aged 30, of light hair and complexion, of medium stature and build, with a full and rounded chest—had never been of a robust constitution, nor able to perform much labor without complaining of excessive fatigue; still never felt the necessity of using medicine. At the time Dr. W. first saw him, he complained of a pain in the chest, and located it two inches above, and one to the right of the right nipple, which was mitigated slightly by the use of remedies; and in about 2 weeks described it as having entered through to, and under the right clavicle. He (Mr. A.) considered the disease of no importance—that it would yield readily to slight treatment. Dr. W., on the contrary, looked upon it as a grave matter, and used energetic means to subdue the disease, which he considered was of the lungs; forming his diagnosis from the use of the stethescope, which spoke in language too plain to be misunderstood. The pain continued slightly, without coughing, expectoration, diarrhœa or night sweats, until the 20th June, '49 when he expired without any of the symptoms peculiar to *phthisis pulmonalis*. His treatment consisted at first of antimony, counter-iritants to the chest and spine as blisters, cupping, and the use of moxa followed by proto iodid Hyd. succeeded by Iod. of Potass and opiates without any beneficial effects whatever. Some five weeks prior to his death, I located in this place, and became associated with Dr. W. in the practice of medicine, at which time we saw him, (Dr. W. had not seen him for two weeks on account of illness) We found him laboring under complete paraplegia, including the pelvic region, which to us was a matter of much interest in ascertaining the cause of this paralytic attack. We were well satisfied that his disease was of the lungs. "Auscultation told the tale." There was no soreness or tenderness on pressure over the region of the spine; and having been taught that if the immediate cause of abolished nervous function was seated in the uppei portion of the spine, or nerves of that part, paralysis would have attacked the superior extremities, consequently we were at a loss to account for the paraplegia, and I must confess that I am not yet satisfied relative to this matter. Sensibility and the power of motion were both destroyed. It was found necessary to evacuate the bladder daily until his death, by use of the catheter. His treatment from this time on, consisted simply in remedies calculated to ease him gently down the declivity of time, as we were well satisfied that he must die. On auscultation we found over the region of the superior lobe of the right lung, no perceptible sound for a space of 2 inches in diameter; the other parts of the lungs gave forth a peculiar low whizzing sound, much like blowing gently into an empty gallon tinct. bottle. Such has been the history of the case.

Post mortem 9 hours after death. Present, Drs. Crain and Marsh. I found the lungs, except the specimen sent, of a dark brown color, much softened—could be easily torn with the fingers—filled with a light red serous substance, and frothy. On the inferior lobe of the left lung, at its base, was a spot the size of a shilling, of a hard cheesy substance, supposed to be the origin of an osseous formation similar to the specimen sent you, which was taken from the superior lobe of the right lung, firmly adherent to the pleura and attached to the spine. It is to be much regretted that we did not make a more thorough dissection; but not expecting to find disease of this nature—making examination to satisfy his friends, and for our own defence as physicians, as others denied the existence of disease of the lungs, is our only excuse.

### REMARKS.

The specimen sent was a piece of lung, in which was imbedded a mass of calcareous matter, of a globular form, and more than an inch in diameter, exceedingly firm at its centre, softer upon its surface, and surrounded by a very firm cyst.

It undoubtedly presents an example, one of the most striking on record, of the cure of tubercular disease of the lungs, where the masses had attained a large size. Laennec, Andral, and others, while admitting the existence of such cicatrization, t of rare occurrence; Louis on the other hand, by negative evidence, and his silence on this point, seems to donbt its occurrence.

It would seem that while the evidences of the fatality of tubercle are abundant, the proofs of its curability have been greatly neglected, and when found, have only presented themselves accidentally. The frequency with which they have been met with, is calculated greatly to encourage us in efforts to relieve consumption. The late Dr. Parish presented a case of the kind.

In the present instance, the want of a history of the individual, and the circumstances under which the cure took place, and the means used, if any, to favor it, is much to be regretted. We abstain from any comments on the cause of paralysis, not being in possession of any facts calculated to throw light on that point, but present our thanks to Drs. Williams and Johnson, for the valuable specimen, which will be carefully preserved. D. B.

